# Astrocytes Differentiated from LRRK2-I1371V Parkinson’s-Disease-Induced Pluripotent Stem Cells Exhibit Similar Yield but Cell-Intrinsic Dysfunction in Glutamate Uptake and Metabolism, ATP Generation, and Nrf2-Mediated Glutathione Machinery

**DOI:** 10.3390/cells12121592

**Published:** 2023-06-08

**Authors:** Roon Banerjee, Aishwarya Raj, Chandrakanta Potdar, Pramod Kumar Pal, Ravi Yadav, Nitish Kamble, Vikram Holla, Indrani Datta

**Affiliations:** 1 Department of Biophysics, National Institute of Mental Health and Neurosciences, Institute of National Importance, Bengaluru 560029, Karnataka, India; 2 Department of Neurology, National Institute of Mental Health and Neurosciences, Institute of National Importance, Bengaluru 560029, Karnataka, India

**Keywords:** mutations in LRRK2 GTPase domain, glutamate uptake and metabolism, glutamate dehydrogenase, glutamate transporters, glutamate to glutamine, glutathione, Nrf2-regulated antioxidants

## Abstract

Owing to the presence of multiple enzymatic domains, LRRK2 has been associated with a diverse set of cellular functions and signaling pathways. It also has several pathological mutant-variants, and their incidences show ethnicity biases and drug-response differences with expression in dopaminergic-neurons and astrocytes. Here, we aimed to assess the cell-intrinsic effect of the LRRK2-I1371V mutant variant, prevalent in East Asian populations, on astrocyte yield and biology, involving Nrf2-mediated glutathione machinery, glutamate uptake and metabolism, and ATP generation in astrocytes derived from LRRK2-I1371V PD patient iPSCs and independently confirmed in LRRK2-I1371V-overexpressed U87 cells. Astrocyte yield (GFAP-immunopositive) was comparable between LRRK2-I1371V and healthy control (HC) populations; however, the astrocytic capability to mitigate oxidative stress in terms of glutathione content was significantly reduced in the mutant astrocytes, along with a reduction in the gene expression of the enzymes involved in glutathione machinery and nuclear factor erythroid 2-related factor 2 (Nrf2) expression. Simultaneously, a significant decrease in glutamate uptake was observed in LRRK2-I1371V astrocytes, with lower gene expression of glutamate transporters SLC1A2 and SLC1A3. The reduction in the protein expression of SLC1A2 was also directly confirmed. Enzymes catalyzing the generation of γ glutamyl cysteine (precursor of glutathione) from glutamate and the metabolism of glutamate to enter the Krebs cycle (α-ketoglutaric acid) were impaired, with significantly lower ATP generation in LRRK2-I1371V astrocytes. De novo glutamine synthesis via the conversion of glutamate to glutamine was also affected, indicating glutamate metabolism disorder. Our data demonstrate for the first time that the mutation in the LRRK2-I1371V allele causes significant astrocytic dysfunction with respect to Nrf2-mediated antioxidant machinery, AT -generation, and glutamate metabolism, even with comparable astrocyte yields.

## 1. Introduction

LRRK2 is an intricate protein with several domains including armadillo repeats (ARMs), ankyrin repeats (ANKs), leucine-rich repeats (LRRs), Ras of complex proteins (Roc), C-terminal of Ros (COR), kinase domain, and WD40 repeats [1,2]. The G2019S mutation, which is present in the kinase domain, has been the most extensively researched, and it is the most common mutation in the Caucasian population. However, mutations in the GTPase domain (R1441C/G/H, Y1699C, I1371V) remain relatively less explored [3,4], and have higher incidence in the eastern hemisphere [5,6,7,8,9].

PD-causing LRRK2 mutations represent a gain of function through the direct activation of kinase activity; mutations in the kinase domain lead to an elevation in kinase activity, while mutations in the Roc domain leave LRRK2 in a GTP-bound kinase-active form by disrupting GTP hydrolysis, thus prolonging kinase activity [10,11]. Multiple studies have reported differences in phosphorylation patterns, i.e., autophosphorylation and substrate (Rab10) phosphorylation between kinase and the GTPase domain mutations [12,13]. Drug-response differences between the mutations of kinase and the GTPase domain have been reported clinically [14] and replicated in the dopaminergic (DA) neurons differentiated from the iPSCs of PD patients carrying these mutations [15]. SH-SY5Y cell lines overexpressing these variants of LRRK2 mutations show differential susceptibility between the kinase and GTPase domains [16]. In contrast to the findings in LRRK2 G2019S PD iPSCs-derived DA neurons [17], an impairment in the development of DA neurons with respect to yield was observed in the iPSCs of LRRK2-I1371V PD patients [18]. These studies highlight the need for studying the function and commitment of other cell types carrying the GTPase domain mutation as well.

LRRK2 is not restricted to DA neurons: its expression is also seen in astrocytes [19,20]. Healthy astrocytes are essential for the appropriate maintenance of adjacent neurons. Autopsy data from PD patients have shown the presence of dystrophic reactive astrocytes. Reactive astrocytosis can lead to adverse effects on DA neurons through increased oxidative stress, decreased neurotrophic factor release, increased inflammation, or enhanced glutamate release, leading to excitotoxicity [21,22,23,24,25]. It has been shown that glutamate excitotoxicity leads to the loss of DA neurons and simultaneous motor dysfunction in PD [26,27], highlighting the function of healthy niche cells. Astrocytes play a key function in the removal of glutamate or other neurotransmitters from the synaptic cleft, preventing excitotoxicity and converting glutamate to glutamine to provide the supply back to neurons. In addition, DA neurons, being highly vulnerable to oxidative stress [28], have, in general, significantly less antioxidizing substrate glutathione (GSH) to tackle increased oxidative stress [29,30]. Astrocytes have an advantage over DA neurons in the synthesis of GSH because they possess the capability to use a broader variety of precursor substrates, including glutamate, to generate GSH [31]. Unlike reports on diseases that are neurodegenerative in nature involving basal ganglia, such as multiple system atrophy and supranuclear palsy, postmortem studies of PD patients have reported lower levels of reduced glutathione in the substantia nigra (SN [32]). Researchers have now involved patient-derived cells to understand whether this impairment of glutamate uptake and metabolism or glutathione content is a cell-intrinsic factor in astrocytes derived from PD patients carrying familial mutations or whether this is due to oxidative stress [33] or extracellular α-synuclein aggregates [23] present during disease progression.

Studies of astrocytes differentiated from LRRK2 G2019S PD iPSCs have reported lysosomal impairments, inflammation, and metabolomic changes [34,35,36], with no changes in glutamate uptake in the LRRK2 G2019S PD iPSC-differentiated astrocytes [35,37]. For the mutant variants of the GTPase domain of LRRK2, there are no studies on astroglial biology. Given the striking differences noted in GTPase domain mutations in the properties of DA neurons, it is important to study the astrocytic functions for this mutation as well.

The heterogeneity of PD is now firmly implicated in the differences in drug responses, which have led to the failure of multiple clinical trials [38], in which ethnicity-based differences have been a key component [39,40,41]. Additionally, as the brain tissue affected in PD is seldom available before the autopsy, patient-derived iPSCs are increasingly considered the best store for creating cell-based platforms for conducting research on the brain cells of interest [39,40,42]. Potential drugs can also be studied and tested on a patient-specific astrocyte base, opening up the possibility for personalized medicine. Furthermore, it has been shown that astrocytes in culture replicate the gene expression, signaling, metabolism, potassium uptake, and glutamine–glutamate cycle flux observed in brain astrocytes [43,44].

In this study, we aimed to assess whether LRRK2-I1371V brings about cell-intrinsic alterations to the astroglial biology involving glutamate uptake and metabolism and Nrf2-mediated glutathione machinery and ATP generation using astrocytes derived from LRRK2-I1371V PD-patient iPSCs and LRRK2-I1371V-overexpressed U87 cells. Astrocytes were derived from iPSCs using a two-step differentiation method, and the yield of astrocytes differentiated at the progenitor and the matured state were assessed.

## 2. Materials and Methods

### 2.1. Reagents

From Sigma-Aldrich (St. Louis, MI, USA), we purchased mitomycin C, dibutyryl cyclic-AMP sodium salt (dbcAMP), paraformaldehyde (PFA), SLC1A2 and CD11b antibody, GDH enzyme, and nicotine adenine dinucleotide (NAD). From Thermo Fisher Scientific (Waltham, MA, USA), we purchased KnockOut™ serum replacement (KOSR), Glutamax™, penstrep solution, NEAA solution, β-mercaptoethanol, DMEM/F12, Geltrex™ matrix, StemFlex™ medium, PSC Neural Induction media, N-2 Supplement, Advanced DMEM/F12 media, SSEA4, A2B5, NF1A, GFAP antibody MAP2 antibody DAPI, TRIzol^®^, V5 tag antibody, and an ATP determination kit. From Immunotools, we purchased bFGF, EGF, CNTF and neuregulin. From Polyplus Transfection^®^ (Illkirch, France), we used jetPRIME™ DNA transfection reagent. From GBioscinces (St. Louis, MI, USA), we purchased Blasticidin. We bought BSA, triton-X, tween-20, and agarose from Himedia (Maharashtra, India). The Abcam (Waltham, MA, USA) antibodies purchased included Nanog, Oct4, Tra-1-60, nestin, musashi12, S100β, aquaporin 4 (AQP4), MRP1 and β-actin antibody, Alexa Fluor^®^488 conjugated secondary antibody, Alexa Fluor^®^647 conjugated secondary antibody, Alexa Fluor^®^405 conjugated secondary antibody, and horse radish peroxidase (HRP) conjugated secondary antibody. From BD Biosciences (Franklin Lakes, NJ, USA), we purchased Vimentin antibody. From R&D Systems (Minneapolis, MN, USA), we bought O4 antibody. From Takara (Shiga, Japan), we obtained PrimeScript™ RT Reagent Kit and EmeraldAmp^®^ GT PCR Master Mix. We purchased a SensiFAST™ SYBR^®^ Lo-ROX Kit (Meridian Bioscience, Newtown, OH, USA), Glutamine/Glutamate-Glo™ Assay kit (Promega, Fitchburg, WI, USA), Glutamic Acid Colorimetric Assay Kit (Elabscience Biotechnology Inc., Houston, TX, USA), QuantiChrom™ Glutathione assay kit (BioAssay Systems, Hayward, CA, USA), and Nrf2 antibody (Santa Cruz Biotechnology, Inc., Houston, TX, USA). The Research Resource Identifiers (RRIDs) and other details of all the antibodies used are mentioned in the Supplementary Materials Table S1.

### 2.2. Ethics Clearance

The generation, differentiation, and utilization of hiPSCs in this study were approved by the Institutional Committee for Stem Cell Research (IC-SCR) with IC-SCR no. SEC/05/030/BP.

All animal experiments were performed as per the guidelines set by the Committee for the Purpose of Control and Supervision of Experiments on Animals (CPCSEA), Government of India. These experiments were approved by the Institutional Animal Ethics Committee (IAEC) of the National Institute of Mental Health and Neuro Sciences (NIMHANS) with IAEC reference no. AEC/70/455/B.P. The animals were provided with an advanced environment with a 12:12 h light/dark cycles and food and water as required.

### 2.3. Generation of Neural Progenitors (NPs) from Induced Pluripotent Stem Cells (iPSCs)

Five clones of previously reported iPSC lines for HC (NIMHAi006-A) and LRRK2-I1371V PD (NIMHi001-A) were used in this study [9,45]. Mouse embryonic fibroblast cells inactivated by mitomycin C on gelatin-coated dishes were used as the feeder layer for the iPSCs maintenance. The media comprised DMEM/F12 supplemented with 20% KOSR, 1× Glutamax™, 1× penstrep solution, 1× NEAA, 0.1 mM β-mercaptoethanol, and 20 ng/mL bFGF. The cells were maintained at 37 °C with 5% carbon dioxide (CO_2_), with a change in media every day. When the iPSC colonies attained the appropriate size, these colonies were manually picked up and plated on Geltrex™-coated dishes with a feeder-free iPSC medium consisting of StemFlex™ basal medium supplemented with 10% StemFlex™ supplement, 1× Glutamax™, and 1× penstrep solution. For neural induction, 24 h after plating the iPSC colonies, the feeder-free iPSC medium was replaced with PSC neural induction medium and maintained with a media change every other day [45,46]. After seven days, a neural expansion medium (equal proportion of neurobasal medium and advanced DMEM/F12 supplemented with 2× neural induction supplement, 1× Glutamax™ and 1× penstrep solution) were provided to the cells for the expansion of NPs.

### 2.4. Differentiation of Astrocytes from NPs

Astrocyte differentiation was performed in two steps, as previously reported [47,48]. In the first step, NPs were exposed to astrocyte priming media consisting of neurobasal medium supplemented with 1× N-2 supplement, 1× Glutamax™, 1× penstrep solution, 10 ng/mL EGF, and 10 ng/mL bFGF. The resulting population was positive for NF1A and vimentin, the two typical markers of glial progenitor cells (GPCs). After four days in priming medium, GPCs were provided with terminal differentiation medium, consisting of neurobasal medium supplemented with 1× N-2 Supplement, 1× Glutamax™, 1× penstrep solution, 10 ng/mL CNTF, 10 ng/mL neuregulin 1β, and 0.1 µM db-cAMP for an additional three days to produce a population of terminally differentiated astrocytes.

### 2.5. U87 Astrocyte Cell Line Maintenance

The malignant glioma cell line U87 MG was a gift from Dr. Nandakumar DN, Department of Neurochemistry, NIMHANS. These cells were maintained in DMEM/F12 medium supplemented with 10% FBS, 1× Glutamax™, and 1× penstrep solution.

### 2.6. Plasmid Constructs and Transfection

pDEST51-LRRK2-I1371V (IV) was a gift from Mark Cookson (Addgene plasmid # 29399; RRID:Addgene_29399). This plasmid had a V5 tag on it. Empty vector (EV) was generated using Hind III restriction enzyme, excising the pDEST51-LRRK2-I1371V plasmid at 291 and 6841 base pairs in the LRRK2 region. The U87 cells were transfected with the EV and IV plasmids using the jetPRIME™ DNA transfection reagent. Transfected cells were selected against 1.5 µg/mL blasticidin, and the efficiency of the transfection was measured by quantifying the V5 tag via flow cytometry (Supplementary Figure S1A,B). The LRRK2 expression in EV and IV transfected cells was determined via Western blotting (Supplementary Figure S1C,D).

### 2.7. Phase Contrast Microscopy

Plated cells were observed under an Olympus CKX41 phase contrast microscope using a 40× objective (LCAch N 40×/0.55 PhP ∞/1/FN22). A minimum of three sets of cells were observed, and at least 10 fields were perceived in each case. The microscope was connected to a MicroPublisher 5.0 RTV camera. Images were captured with the Q Capture Pro7 software interface (Q Capture software, RRID:SCR_014432).

### 2.8. Immunocytochemistry

We fixed 90–95% confluent 12 mm coverslips consisting of adherent cells with 4% PFA. Permeabilization was performed using 1% Triton-X, and 3% BSA was used to block unspecific antigens. The markers of interest were detected using the respective primary antibodies (1:100 concentration). The primary antibodies against Nanog, SSEA4, Oct4, Tra-1-60, nestin, musashi12, A2B5, S100β, vimentin, NF1A, GFAP, AQP4, Nrf2 MAP2, CD11b, O4, and SLC1A2 were used. Fluorescent-dye-tagged (Alexa Fluor^®^488 or Alexa Fluor^®^ 647) secondary antibodies (1:200 concentration) against the respective primary antibodies were used for the fluorescence detection. We used 300 nM DAPI to stain the nuclei. PBS with 0.05% tween-20 was used for the washes between every step. DABCO was used to mount the coverslips on glass slides, and these coverslips were observed under a Leica DMi8 fluorescent microscope using a Semi-Apochromats-40×/0.80 PL FLUOTAR objective. The microscope was connected to the Leica camera. Three sets were processed, and at least 10 fields were observed in each case. Leica Application Suite X (LasX) software version 3.3.3.16958 (Leica Application Suite X, RRID:SCR_013673) was used to process the images.

### 2.9. Flow Cytometry

Astrocytes (1 × 10^5^ cells) were prepared as single-celled suspensions and were fixed with 2% PFA. Permeabilization was performed using 1% triton-X (for internal antigens), and 3% BSA was used to block unspecific antigens. The markers of interest were detected using the respective primary antibodies (1:100 concentration). The primary antibodies against nestin, musashi12, A2B5, S100β, vimentin, NF1A, GFAP, AQP4, MAP2, CD11b, O4, SLC1A2, and MRP1 were used. Fluorescent-dye-tagged (Alexa Fluor^®^488, Alexa Fluor^®^ 647 or Alexa Fluor^®^ 405) secondary antibodies (1:200 concentration) against the respective primary antibodies were used for fluorescence detection. PBS with 0.01% NaN_3_ was used for washing the cells between each step. FACS Verse (BD Biosciences, Franklin Lakes, NJ, USA) was used to measure the cells, and BD FACSuite software was used for the analysis. Light scatter for 10,000 gated events was used to identify cells. Secondary-antibody-stained astrocytes were used for gating (red line) out nonspecific staining and are represented by the shaded peaks in the flow cytometry histograms [23].

### 2.10. Quantitative Polymerase Chain Reaction (qPCR)

TRIzol^®^ reagent was used for the extraction of RNA from the astrocytes. A PrimeScript™ RT Reagent Kit was used to synthesize cDNA from 1µg of the extracted RNA. A SensiFAST™ SYBR^®^ Lo-ROX Kit was used to perform qPCR in a QuantStudio™ 6 Flex Real-Time PCR System (applied biosystems^®^ by life technologies™, Carlsbad, CA, USA). The data obtained are represented as fold changes in the mRNA levels in the astrocytes normalized with the respective *18S* mRNA. The primers are listed in Supplementary Materials Table S2.

### 2.11. Glutathione Content

The reduced glutathione contents in the cell lysates of the astrocytes were quantified using a QuantiChrom™ Glutathione assay kit. An Infinite^®^ 200 Microplate reader (Tecan, Männedorf, Switzerland) was used to obtain the absorbance reading at 412 nm. The glutathione content was determined from the cell lysate obtained from the astrocytes per the manufacturer’s instructions using the calibrator provided with the kit.

### 2.12. Glutamine and Glutamate Contents

The glutamine and glutamate contents in the cell lysates of the astrocytes were evaluated using a Glutamine/Glutamate-Glo™ Assay per the manufacturer’s instructions, via luminescence detection. This kit is based on two steps: first, the conversion of glutamine to glutamate by the glutaminase enzyme; second, glutamate detection. Thus, both glutamine and glutamate levels were detected in separate wells. The luminescence emitted was obtained using an Infinite^®^ 200 Microplate reader (Tecan).

### 2.13. Glutamate Uptake

The protocol procedure was similar to that of earlier studies [23,49]. Briefly, astrocytes were treated with 100 mM glutamate for 2 h, and their glutamate contents were assessed using a Glutamic Acid Colorimetric Assay Kit per the manufacturer’s instructions. The glutamate levels in the astrocytes derived from the PD iPSCs were compared with those of the astrocytes derived from the PD iPSCs not exposed to extracellular glutamate (C1) and with those of healthy astrocytes exposed to 100 mM glutamate in the extracellular medium (C2). The optical density was detected at 340 nm using an Infinite^®^ 200 Microplate reader (Tecan).

### 2.14. ATP Determination

ATP was quantitatively determined in astrocytes with a bioluminescence assay using an ATP determination kit, as per the manufacturer’s instructions. This is a highly sensitive reaction that detects the ATP in the cell lysate obtained from 20,000 cells per reaction. The luminescence emitted was determined using an Infinite^®^ 200 Microplate reader (Tecan).

### 2.15. Time-Lapse Live-Cell Fluorescence Imaging for Glutamate Dehydrogenase (GDH) Enzyme Kinetics

The reversible glutamate to α-ketoglutarate reaction is facilitated by the enzyme GDH along with the NAD/NADH redox reaction. The conversion of glutamate to α-ketoglutarate, with the reduction of NAD to NADH, emits fluorescence when excited with UV light (NADH). The GDH enzyme kinetics were studied using a previously reported procedure [23,50]. Astrocytes on a 22 mm coverslip were immersed in Hank’s balanced salt solution (HBSS, pH 7.4), with the addition of GDH (76 I.U./mL) and NAD (1 mM). Furthermore, the live-cell fluorescence intensity was recorded every 1 s for 500 s under a Leica DMi8 fluorescence inverted microscope with a Leica DFC3000G camera supported with LAS X image acquisition software version 3.3.3.16958 (Leica Application Suite X, RRID:SCR_013673). ImageJ software version 1.52f (ImageJ, RRID:SCR_003070) was used to analyze the data. All imaging data are expressed as F1/F0, and the area under the curve was plotted.

### 2.16. Sodium Dodecyl Sulfate–Polyacrylamide Gel Electrophoresis (SDS-PAGE) and Western Blot

We used 40 μg of the protein extracted from the cell culture lysate, as shown in our earlier publications [23,51]. This was separated by SDS-PAGE. The semidry transfer method was used to transfer the separated protein onto a polyvinyl difluoride (PVDF) membrane, followed by blocking the unspecific antigens with 5% BSA and probing the membranes using primary antibodies at a 1:1000 concentration. The primary antibodies against LRRK2, β-actin, Nrf2, and SLC1A2 were used. Then the membranes were incubated with HRP-conjugated secondary antibody at a 1:2000 concentration. Tris-buffered saline with 0.1% Tween-20 was used for washing the membranes between each step. The substrate for HRP was SuperSignal^®^ West Pico Chemiluminescent Substrate. A ChemiDoc™ MP Imaging System (BioRad) was used to observe the bands. Densitometric analysis for Nrf2 was normalized for β-actin and is presented as relative density. ImageJ software version 1.52f (ImageJ, RRID:SCR_003070) was used to analyze these data. 

### 2.17. Statistical Analysis

All the results are reported as mean ± standard deviation (SD). A two-sample *t*-test, calculated using R software version 3.4.1 (R Foundation; R Project for Statistical Computing, RRID:SCR_001905), was used to obtain the statistical comparisons. A *p*-value less than 0.05 was considered significant. For statistical significance, * represents *p* < 0.05, ** represents *p* < 0.01, and *** represents *p* < 0.001. Graphs were prepared using GraphPad Prism 6 (GraphPad Software; GraphPad Prism, RRID:SCR_002798).

## 3. Results

### 3.1. Generating Terminally Differentiated Astrocyte Populations from iPSC Lines

**Figure 1:**
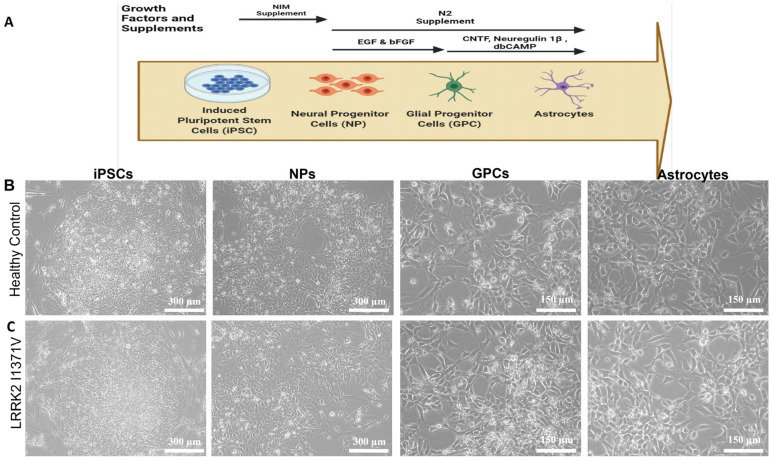
Differentiation of iPSCs into astrocytes: (**A**) Diagrammatic representation of differentiation of astrocytes from iPSCs. The iPSCs were differentiated into neural progenitor (NP) cells from which glial progenitor cells (GPC) were generated. The GPCs were then differentiated into astrocytes. (**B**,**C**) Representative phase contrast images for the healthy control (**B**) iPSC line and the patient-derived iPSC line carrying the PD LRRK2-I1371V mutation (**C**) at different stages of differentiation.

**Figure 2:**
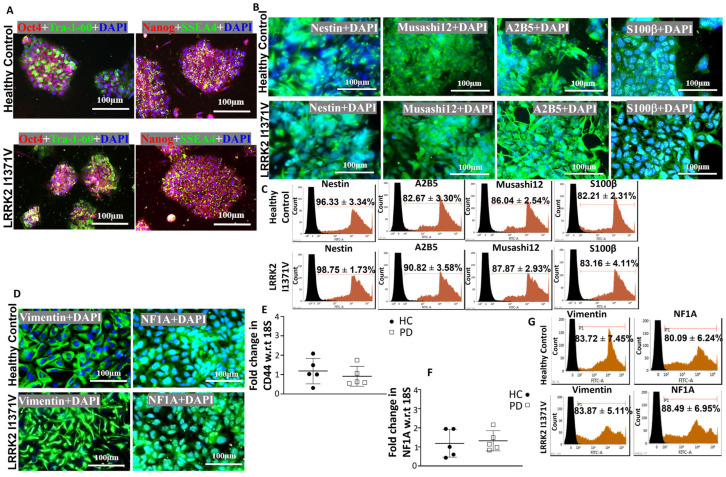
Generation of GPCs from iPSC lines: (**A**) ICC images of healthy control (HC) and Parkinson’s disease LRRK2-I1371V (PD) iPSCs for pluripotency markers Oct4, Tra-1-60, Nanog, and SSEA4. (**B**) ICC images of neural progenitor cells (NPs) from the healthy control and the LRRK2-I1371V iPSC line staining positive for NP cell markers nestin, musashi12, A2B5, and S100β; magnification 40×. (**C**) Flow cytometry histogram of NPs differentiated from the HC and PD iPSC lines immunolabeled with nestin, A2B5, musashi12, and S100β. (**D**) ICC images of glial progenitor cells (GPCs) from HC and PD iPSCs staining positive for GPC markers vimentin and nuclear factor 1A (NF1A). (**E**,**F**) qPCR analysis of GPC markers CD44 (**E**) and NF1A (**F**) of GPCs derived from HC and PD iPSCs. (**G**) Flow cytometry histogram of GPCs immunolabeled with vimentin and NF1A. The nuclei in the ICC images were counterstained with DAPi. *n* = 5; mean ± SD; w.r.t, with respect to.

**Figure 3:**
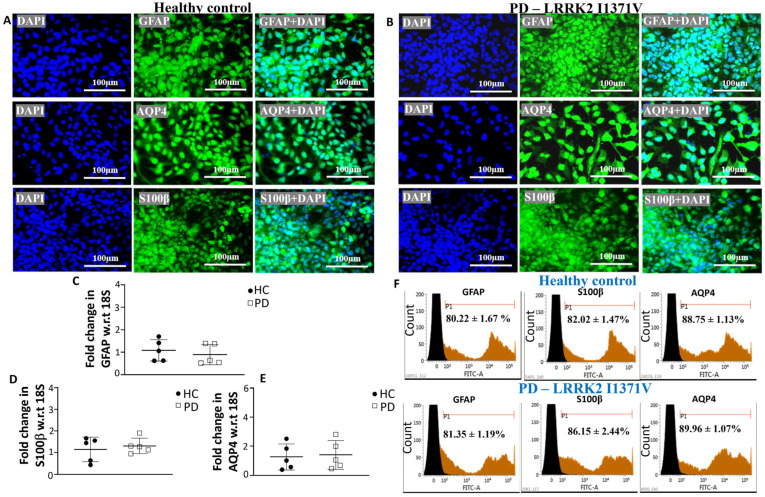
Characterization of astrocytes derived from HC and PD LRRK2-I1371V GPCs: (**A**,**B**) ICC images of astrocytes from HC (**A**) and PD (**B**) iPSC lines staining positive for mature astrocyte markers GFAP, AQP4, and S100β. The nuclei were counterstained with DAPI. (**C**,**E**) qPCR analysis of mature astrocytes markers GFAP (**C**), S100β (**D**), and AQP4 (**E**) of mature astrocytes derived from HC and PD iPSCs; w.r.t, with respect to. (**F**) Flow cytometry histograms of astrocytes differentiated from the HC and PD iPSC lines immunolabeled with GFAP, S100β and AQP4. *n* = 5; mean ± SD.

Astrocytes were differentiated from iPSCs in three stages, as shown in Figure 1A; Figure 1B,C shows the morphological transformations observed during the different stages of astrocyte differentiation. The iPSC lines were fully characterized [9,45], and they expressed pluripotency markers Oct4, Tra-1-60, Nanog, and SSEA4 (Figure 2A). At the NP stage, the cells exhibited both neural progenitor markers nestin and musashi12 as well as early glial progenitor markers A2B5 and S100β (Figure 2B). FACS analysis showed similar immunopositive populations of NPs expressing nestin, A2B5, musashi12, and S100β (Figure 2C). These NPs after priming with EGF and FGF resulted in GPCs expressing vimentin and NF1A (Figure 2D). The GPC identity was further confirmed by quantifying the gene expressions of *CD44* and *NF1A*. However, the mRNA levels were similar between the healthy control (HC) and LRRK2-I1371V (PD) astrocytes (*p* > 0.05 Figure 2E,F). FACS analysis of GPCs showed a >80% immunopositive population for vimentin and NF1A (Figure 2G). The subsequent differentiation for another three days in the presence of CNTF, neuregulin 1β, and db-cAMP resulted in a population of terminally differentiated astrocytes that showed robust expressions of GFAP, AQP4, and S100β (Figure 3A,B). The gene expressions of *GFAP*, *S100β*, and *AQP4* were similar between the healthy control (HC) and LRRK2-I1371V (PD) astrocytes (*p* > 0.05; Figure 3C–E). FACS analysis showed that astrocytic differentiation yielded a similar immunopositive population of terminally differentiated astrocytes expressing 80% of terminal astrocyte markers such as GFAP, S100, and AQP4 (Figure 3F). No expression of mature neuronal marker MAP2, oligodendrocytes marker O4, or microglial marker CD11b was observed (Supplementary Figure S2A–C). This suggested that both the iPSC lines were able to generate terminally differentiated astrocytes, and there was no difference in the yield of astrocytes between healthy control (HC) and LRRK2-I1371V (PD) iPSCs.

### 3.2. Glutathione Machinery

**Figure 4:**
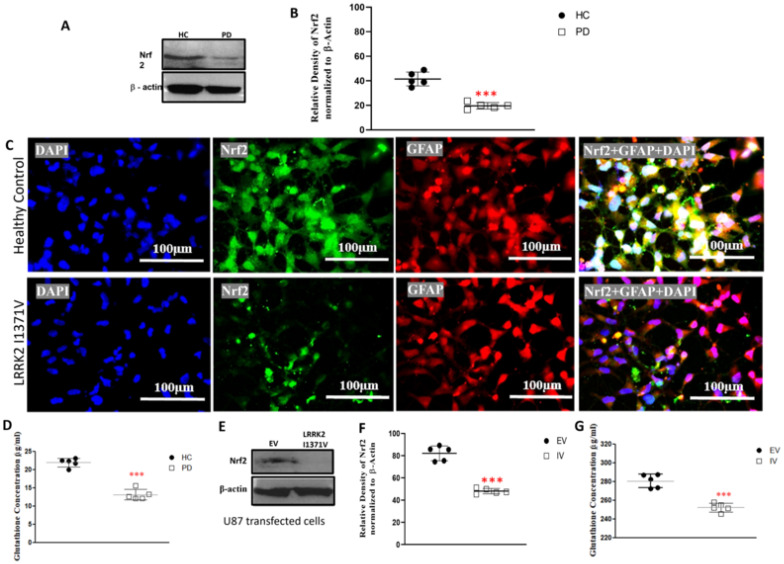
Expression of nuclear factor erythroid-2-related factor 2 (Nrf2) and reduced glutathione concentration: (**A**) Western blot analysis using antibodies targeting Nrf2 and β-actin (HC, healthy control; PD, Parkinson’s disease LRRK2-I1371V). (**B**) Densitometric analysis of the bands obtained from Western blotting. (**C**) ICC images of GFAP (Alexa Fluor^®^ 647; red)-marked HC and PD astrocytes, expressing Nrf2 (Alexa Fluor^®^ 488; green) and nuclei counterstained with DAPI. (**D**) Reduced glutathione content in HC and PD astrocytes. (**E**) Western blot analysis using antibodies targeting Nrf2 and β-actin (EV, U87 cells transfected with EV; IV, U87 cells transfected with LRRK2-I1371V). (**F**) Densitometric analysis of the bands obtained from Western blotting. (**G**) Reduced glutathione content in EV and IV transfected U87 cells. *n* = 5; mean ± SD; ***—*p* < 0.001 (HC versus PD or EV versus IV).

**Figure 5:**
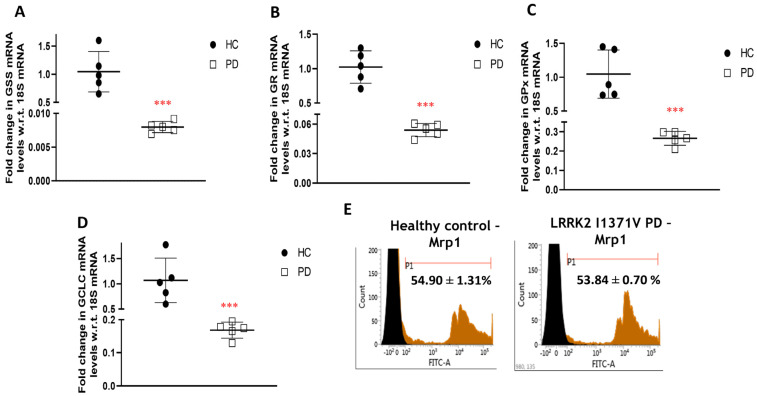
Expression of glutathione-related genes and proteins: (**A**–**D**) qPCR analysis of (**A**) *glutathione synthetase* (*GSS*), (**B**) *glutathione reductase* (*GR*), (**C**) *glutathione peroxidase* (*GPx*), and (**D**) *glutamate-cysteine ligase* (*GCLC*); w.r.t, with respect to. (**E**) Flow cytometry histogram of multidrug-resistance-associated protein (MRP-1) in HC and PD astrocytes. *n* = 5; mean ± SD; ***—*p* < 0.001 (HC versus PD).

Nrf2 functions as the key transcription factor responsible for the activation of a plethora of cytoprotective genes, some of which include key components of the glutathione antioxidant system. Western blot analysis revealed a significantly lower expression of Nrf2 in LRRK2-I1371V astrocytes in comparison with that of the HC (*p* < 0.001; Figure 4A,B). A distinct nuclear localization of Nrf2 expression was observed in the astrocytes derived from HCs in contrast with that of the LRRK2-I1371V via immunofluorescence (Figure 4C). The corresponding glutathione (GSH) level was distinctly lower in LRRK2-I1371V astrocytes than in HCs (*p* < 0.001; Figure 4D). A similar reduction in the expression of Nrf2 was observed in U87 cells transfected with LRRK2-I1371V (IV) in comparison with that in U87 cells transfected with EV (*p* < 0.001; Figure 4E,F), with a corresponding reduced level of GSH (*p* < 0.001; Figure 4G). The expression of GSH-related enzymes *glutathione synthetase* (*GSS*), *glutathione reductase* (*GR*), and *glutathione peroxidase* (*GPx*) at mRNA level was also significantly lower in LRRK2-I1371V astrocytes (*p* < 0.001; Figure 5A–C). The de novo synthesis of GSH is catalyzed not only by GSS in an AT- dependent manner but also by glutamate cysteine ligase (GCL); its catalytic subunit being *GCLC*, which also showed reduced expression in LRRK2-I1371V astrocytes (Figure 5D). The expression of multidrug-resistance-associated protein (MRP1), which acts as a glutathione transporter, however, was similar between the HC and LRRK2-I1371V astrocytes (*p* > 0.05; Figure 5E).

### 3.3. Glutamate Uptake and Metabolism

**Figure 6:**
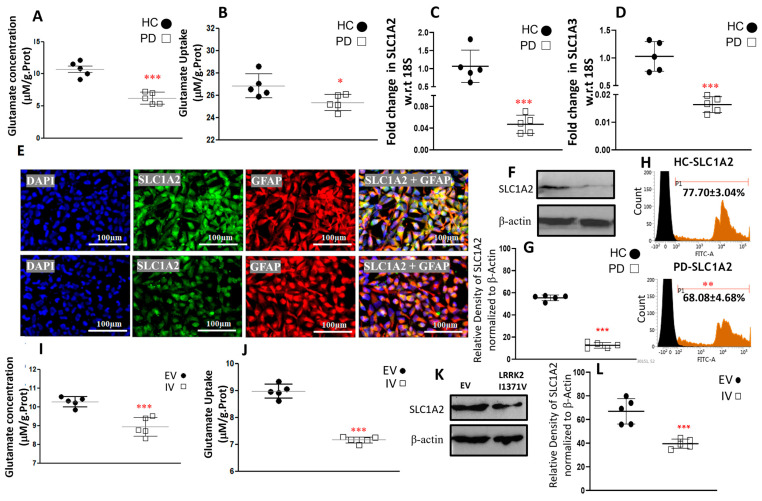
Glutamate content and uptake along with the expression of genes and proteins associated with its uptake. (**A**) Glutamate content in HC and PD astrocytes. (**B**) Glutamate uptake capacity in HC and PD astrocytes. (**C**,**D**) qPCR analysis of mRNA levels of (**C**) *excitatory amino acid transporter 2* (*SLC1A2*) and (**D**) *excitatory amino acid transporter 1* (*SLC1A3*); w.r.t, with respect to. (**E**) ICC images of SLC1A2 (Alexa Fluor^®^ 488; green) and GFAP (Alexa Fluor^®^ 647; red) coimmunostained HC and PD astrocytes; nucleus counterstained with DAPI. (**F**) Western blot analysis using antibodies targeting SLC1A2 and β−actin (HC, healthy control; PD, Parkinson’s disease LRRK2−I1371V). (**G**) Densitometric analysis of the bands obtained via Western blotting. (**H**) Flow cytometry histogram of SLC1A2 in HC and PD astrocytes. (**I**) Glutamate content in EV and IV transfected U87 cells. (**J**) Glutamate uptake capacity in EV and IV transfected U87 cells (**K**) Western blot analysis using antibodies targeting SLC1A2 and β−actin (EV, U87 cells transfected with empty vector; IV, U87 cells transfected with LRRK2−I1371V). (**L**) Densitometric analysis of the bands obtained via Western blotting. *n* = 5; mean ± SD; *—*p* < 0.05 (HC versus PD); **—*p* < 0.01; ***—*p* < 0.001 (HC versus PD or EV versus IV).

Glutamate uptake from the synaptic cleft, and its metabolization to glutamine for supply to neighboring neurons, is a key function of astrocytes. To assess the impact of the LRRK2-I1371V mutation on the various functions related to glutamate, we measured the basal levels of glutamate in HC and LRRK2 I1371V astrocytes. The glutamate content in the HC astrocytes was significantly higher than that in the LRRK2-I1371V astrocytes (*p* < 0.001; Figure 6A). When exposed to 100 µM extracellular glutamate, the uptake of glutamate was significantly compromised in LRRK2-I1371V astrocytes compared with that in HCs (*p* < 0.001; Figure 6B). A corresponding lower mRNA expression of glutamate transporters *SLC1A2* and *SLC1A3* was also detected in the LRRK2-I1371V astrocytes than in the HCs (*p* < 0.001; Figure 6C,D). Immunofluorescence displayed a lower expression of SLC1A2 in the astrocytes derived from the LRRK2-I1371V iPSCs in comparison with that in the HCs (Figure 6E). Western blot analysis showed significantly reduced protein expression of SLC1A2 in the LRRK2-I1371V astrocytes in comparison with that in the HCs (*p* < 0.01; Figure 6F,G), which was further confirmed by flow cytometry analysis (*p* < 0.01; Figure 6H). The glutamate content in the LRRK2-I1371V transfected U87 cells was significantly lower than that in the EV transfected U87 cells (*p* < 0.001; Figure 6I). Furthermore, distinctly reduced uptake of glutamate was observed in LRRK2-I1371V transfected U87 cells in comparison with that in EV transfected ones in the presence of 100 μM extracellular glutamate (*p* < 0.001; Figure 6J). Here too the expression of SLC1A2 was observed to be significantly lower in the LRRK2-I1371V transfected U87 cells (*p* < 0.001; Figure 6K,L).

**Figure 7:**
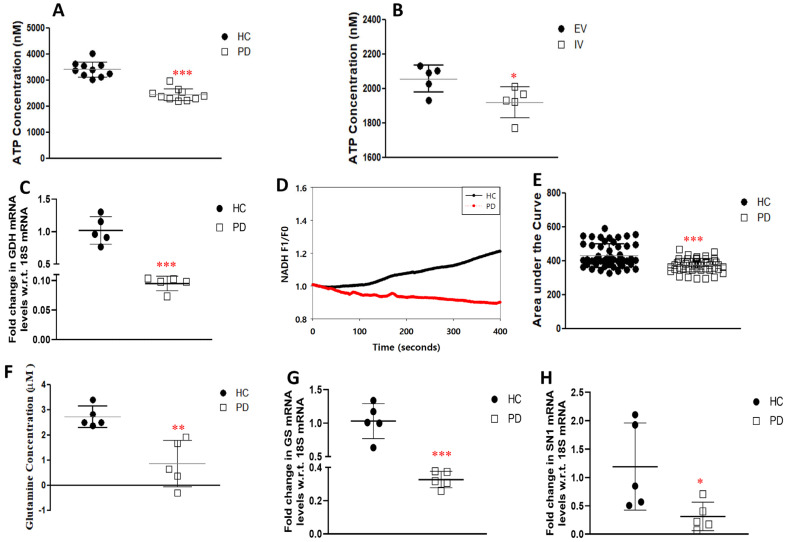
Glutamate metabolism and expression of genes associated with it and its transport. (**A**) Functional ATP production luminescence in HC and PD astrocytes. (**B**) Functional ATP production luminescence in EV and IV transfected U87 cells. (**C**) qPCR analysis of mRNA levels of *glutamate dehydrogenase* (*GDH*). (**D**) Activity of GDH enzyme in HC and PD astrocytes. (**E**) Cumulative F1/F0 is represented as the area under the curve for HC and PD astrocytes. (**F**) Glutamine content in HC and PD astrocytes. (**G**,**H**) qPCR analysis of mRNA levels of (**G**) *glutamine synthase* (*GS*) and (**H**) *system N1 Na^+^ and H^+^—coupled glutamine transporter* (*SN1*). *n* = 5; mean ± SD; w.r.t, with respect to; *—*p* < 0.05 (EV versus IV or HC versus PD); **—*p* < 0.01 (HC versus PD); ***—*p* < 0.001 (HC versus PD).

On uptake, glutamate can enter the TCA cycle under the action of the enzyme GDH and undergo catabolism to produce ATP. The LRRK2-I1371V astrocytes showed distinctly lower ATP production than the HC astrocytes (*p* < 0.001; Figure 7A). In the LRRK2-I1371V transfected U87 cells, a significantly lower level of ATP was observed in comparison with that in the EV astrocytes (*p* < 0.05; Figure 7B). Glutamate is also synthesized de novo from the intermediate product of the TCA cycle, α-ketoglutarate, by GDH. Due to its reversible nature, GDH is an important enzyme in determining the levels of glutamate in astrocytes. *GDH* mRNA expression was significantly lower in LRRK2-I1371V astrocytes than in HCs (*p*< 0.001; Figure 7C). As a confirmatory test, the kinetics of the enzyme GDH was measured through live-cell imaging. NAD, which is a cofactor for GDH, undergoes conversion to fluorescent NADH after GDH activity, and NADH fluorescence is quantified using time-lapse fluorescence imaging [23,47,52]. Upon quantification of the emitted fluorescence by NADH, it was revealed that the activity was increased in the HC astrocytes compared with that in LRRK2-I1371V cells (*p* < 0.001; Figure 7D,E).

**Figure 8:**
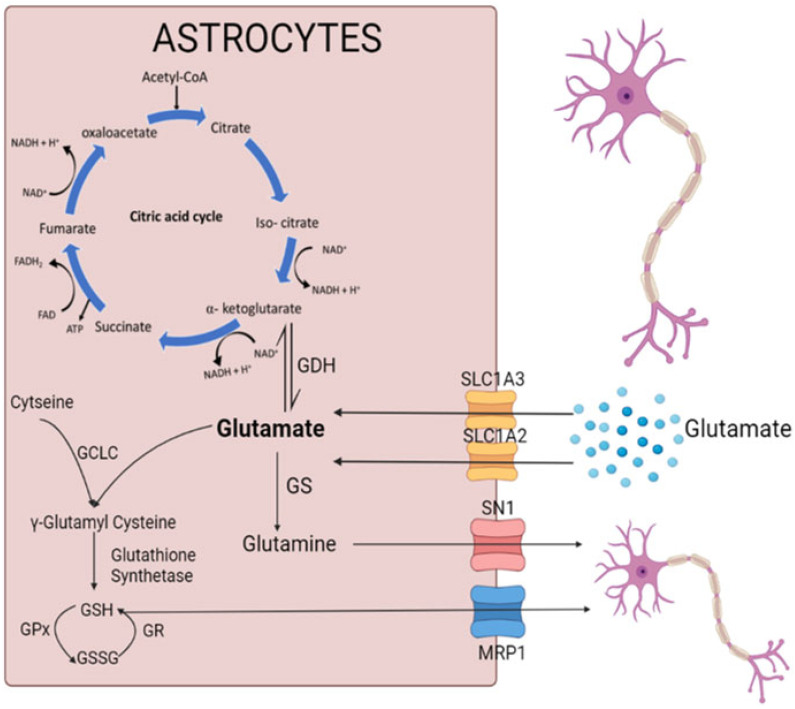
The schematic represents the link between the glutamate uptake to its conversion to glutamine, generation of γ glutamyl cysteine (precursor of glutathione), and metabolism to enter Kreb’s cycle (α-ketoglutaric acid). Created with Biorender.com.

Astrocytes are the sole sites responsible for the de novo synthesis of glutamine, which involves the activity of the GS enzyme converting glutamate to glutamine. The formed glutamine is then transported out of the astrocytes to neurons via the system N1 Na^+^ and H^+^-coupled glutamine transporter (SN1) channel. The glutamine content in LRRK2-I1371V astrocytes was significantly lower than in the HC astrocytes (*p* < 0.01; Figure 7F). In addition, the gene expression of the *GS* as well as the *SN1* showed a significant reduction in the PD astrocytes compared with that in the HC astrocytes (*p* < 0.001 and *p* < 0.05 respectively; Figure 7G,H). Figure 8 schematically depicts the link between the glutamate uptake to its conversion to glutamine, generation of γ glutamyl cysteine (precursor of glutathione), and metabolism to enter the Kreb’s cycle (α-ketoglutaric acid).

## 4. Discussion

LRRK2 mutant variants show distinct topographical distributions, e.g., G2019S is predominant in familial PD patients in Caucasian populations [53] and Ashkenazi Jews (AJ) [54], whereas R1441C/G, I1371V, R1628P, and G2385R are more common among east Asian PD patients [55,56]. Furthermore, differences in disease severity (tremor and motor fluctuations) [5,57,58], age of onset [59], brain pathology [60,61,62], and drug response [63,64] have been reported among LRRK2 variants. LRRK2 phosphorylates not only itself (autophosphorylation) but also Rab proteins, which serve as its primary substrate [65,66]. The differences in substrate phosphorylation patterns of the different mutant variants of LRRK2 have been demonstrated in knock-in mice model and human samples [12,13]. Differences in the α-synuclein phosphorylation pattern in rodent models expressing different variants of LRRK2 (G2019S and R1441C) have also been reported, confirming that there are differences in phosphorylation among LRRK2 variants. Cooper et al. also showed the differences in the effects of two LRRK2 variants G2019S and R1441C on mitochondrial dysfunction and the degree of drug response for the same pharmacological molecules, using an in vitro platform, thus highlighting the differential effects of LRRK2 mutations on cellular functions [15].

In our previous study, we showed that disease pathology is indeed replicated in dopaminergic neurons differentiated from LRRK2-I1371V iPSCs. However, the effect of this mutation on astrocyte biology with respect to the Nrf2-associated antioxidant system as well as glutamate uptake and metabolism has not been studied. The impact of this mutation variant on bringing about cell-intrinsic changes in these key functions of astrocytes will provide evidence of the inherent pathogenicity of this variant on astrocytes, even in the absence of extrinsic stress in the form of ROS or extracellular α-synuclein.

Astrocytes were differentiated from a PD iPSC line carrying the LRRK2-I1371V mutation obtained from a patient with a confirmed diagnosis of PD. The diagnosis was based on the Unified Parkinson’s Disease Rating Scale (UPDRS) score and conclusively verified by ^[18F]^fluoro-L-dopa (F-DOPA) positron emission tomography (F-DOPA PET). The iPSC lines used in this study have been fully characterized and registered with hPSCreg [9,45]. As a further confirmation of the results from astrocytes derived from PD iPSCs, the same assessments of glutathione content and glutamate uptake were independently performed in LRRK2-I1371V transfected U87 cells.

Our earlier study showed that the presence of an optimum number of astrocytes is crucial for the survival of DA neurons under stress [67]. From the comparable yield of the immunopositive population of differentiated cells expressing terminal astrocyte markers AQP4 and GFAP, it is evident that the LRRK2-I1371V mutation did not interfere with the yield/number of astrocytes. However, the expression of Nrf2, the critical regulatory transcription factor for the glutathione antioxidant system, was found to be significantly lower in the LRRK2-I1371V-derived astrocytes. Additionally, the transcription factor was distinctly localized in the nucleus in the HC-derived astrocytes, unlike in the LRRK2 mutation-carrying astrocytes. We reported a similar feature of Nrf2 in healthy astrocytes under stress (exposed to α-synuclein aggregates), along with a weakening of the glutathione antioxidant system [23].

A growing body of evidence suggests that LRRK2 mutations are associated with the PD-induced death of DA neurons by enhancing ROS at the cellular level [68,69]. However, the impact of the LRRK2 GTPase-variant mutation on the cell-intrinsic deregulation of antioxidant by astrocytes is not known. Astrocytes maintain the antioxidant regulation in the CNS through the Nrf2–ARE pathway. Neurons have weak Nrf2 activity and are solely dependent on astrocytes to mitigate oxidative stress [29,30]. A recent study showed significantly reduced Nrf2 expression in LRRK2 transgenic mouse brains and LRRK2-overexpressing SH-SY5Y cells [70]. In the LRRK2-I1371V transfected U87 cells, a similar pattern of lower expression of Nrf2 was observed, suggesting that LRRK2 can influence Nrf2 expression. Being a kinase, LRRK2 has the ability to phosphorylate multiple proteins depending on its activity and duration of activation [71]. In its monomeric form, it binds to the 14-3-3 protein in the cytosol, which further acts as a scaffold for numerous signaling proteins and cellular pathways [72]. With reference to the kinase (G2019S) domain variant of LRRK2, a couple of studies have shown Nrf2-mediated neuroprotection [73,74], but the influence of the mutation on its downstream antioxidants has not been reported. The antioxidant substrate glutathione can exist in two different forms: GSH (the reduced form) and GSSG (the oxidized form) [75]. GSH is formed from gamma glutamyl cysteine (GGC) in an ATP-dependent manner by the enzyme GSS, while GGC is formed from the substrate glutamate upon the action of GCL [76]. GSH is converted to GSSG under the action of GPx, and GSSG is transformed back to GSH under the action of the enzyme GR. The reduced form of glutathione, GSH, is extracellularly released through the MRP1 transporters located on the cell membrane for its uptake by adjacent neurons. In the LRRK2-I1371V PD astrocytes, a distinct reduction in glutathione content was noted in comparison with that in the HC astrocytes, which may have been due to the lower expressions of GSS, GR, and GCLC (catalytic unit of GCL) in the PD astrocytes along with reduced levels of ATP that are needed by GSS. LRRK2-I1371V transfected U87 cells also showed lower glutathione contents, suggesting that LRRK2-I1371V can intrinsically affect this Nrf2-mediated antioxidant system. Thus, we conclude that the LRRK2-I1371V mutation clearly impairs/interferes with the glutathione machinery of the astrocytes.

One of the cardinal functions of astrocytes is the uptake of glutamate from the synaptic cleft via the EAAT2 (SLC1A2) and EAAT1 (SLC1A3) channels, thus regulating extracellular glutamate levels [77], with approximately 90% of glutamate transport being mediated by EAAT2 [78,79,80]. Glutamate dyshomeostasis is a familiar feature of PD, as indicated by clinical reports showing altered glutamate levels in the idiopathic PD patient brain and plasma [81,82]. Furthermore, in vivo studies revealed that glutamate excitotoxicity can lead to DA neuron degeneration in PD [26]. Once inside astrocytes, glutamate has three possible outcomes: (i) it can convert to glutamine under the action of the GS enzyme, (ii) it can enter the TCA cycle by forming α-ketoglutarate (a TCA cycle intermediate) under the action of the enzyme GDH, or (iii) it can form GGC (an intermediate step in the formation of glutathione) under the action of GCL in the presence of cysteine, ultimately leading to the synthesis of reduced glutathione. The extracellular release of glutamine from astrocytes occurs through the SN1 transporter. This glutamine serves as a substrate for the formation of glutamate in the neurons to replenish the neurotransmitter pools [52]. Impairment of glutamate uptake was observed in LRRK2-I1371V astrocytes, which was also reflected by the lower basal glutamate in these cells in comparison with that in the HC iPSC-derived astrocytes. The primary reason for this was the significantly lower expression of glutamate transporters SLC1A2 and SLC1A3 in the LRRK2-I1371V astrocytes. The glutamate uptake feature observed here may be contrasted with those of the studies reported from G2019S iPSC-derived astrocytes. This is possibly due to the differential effect on the substrate (Rab10) phosphorylation between the kinase- and GTPase-domain mutations of LRRK2, which was observed in a knock-in mouse model [13]. Rab10 phosphorylation was found to be higher for the GTPase domain mutation in comparison with that in the kinase domain mutation; however, autophosphorylation of LRRK2 was higher for the kinase domain mutation [13]. Rab10 is a key protein involved in protein trafficking from the Golgi apparatus to the cell membrane [83]. Indeed, controlled glutamate transporter trafficking by Nedd4-2 was shown to be protective in an in vivo PD rodent model [84]. Furthermore, the decreased glutamate uptake and content in LRRK2-I1371V transfected U87 cells showed that this mutation can intrinsically influence glutamate uptake.

The conversion of glutamate to glutamine was also compromised in the LRRK2-I1371V astrocytes, as demonstrated by the glutamine levels. This might have been a consequence of not only the lower basal glutamate content but also the reduced expression of the GS enzyme. The expression of glutamine transporter SN1 was also found to be low in the LRRK2-I1371V astrocytes. In addition, glutamate enters the TCA cycle for ATP release through its conversion to α-ketoglutarate by the enzyme GDH [85]. GDH expression and activity were significantly lower in the LRRK2-I1371V astrocytes, with a corresponding reduction in the ATP levels in the LRRK2-I1371V astrocytes compared with those in the healthy ones. In transfected U87 cells, where a decreased glutamate uptake and content was observed, a reduced ATP was also obtained, confirming that the LRRK2-I1371V mutation has an intrinsic effect on glutamate metabolism.

## 5. Conclusions

The present study established that astrocytes derived from the iPSCs of PD patients carrying the LRRK2-I1371V mutation had a similar yield as those derived from HCs but distinctly displayed a cell-intrinsic impairment of the Nrf2-mediated antioxidant system and glutamate uptake and metabolism, both core functions of astrocytes. The impairment of the glutathione content and glutamate uptake and metabolism in LRRK2-I1371V transfected U87 cells further confirmed that the mutation inherently affects these key glial functions. The susceptibility to LRRK2-I1371V is evident in the PD patients of east Asian origin carrying this mutation [15], and this study indicates that the compromised neuroprotective function of astrocytes may play a crucial role here. The autopsied PD brains of LRRK2-I1371V patients showed Lewy body pathology [86], and LRRK2 transgenic mice for variants of GTPase and kinase domain mutations showed the classical features of PD [87]. While our study found that astrocytes differentiated from LRRK2-I1371V demonstrate clear impairment of glutathione and glutamate biology, there needs to be further in vivo studies performed on this variant. Therapeutics targeting glutamate transporters were reported to reduce astrogliosis [84], and an antagonist of the ionotropic glutamate receptor NMDA (to decrease response to glutamate excitotoxicity) showed improvement in motor symptoms [88] in an in vivo rodent PD model. The current iPSC model used here can serve as a valuable testing platform for studying the impact of such therapeutic strategies on patient-derived astrocytes.

## Data Availability

All data generated or analyzed during this study are included in this published article and its supplementary information files.

## Supplementary Materials

The following supporting information can be downloaded at: https://www.mdpi.com/article/10.3390/cells12121592/s1, Figure S1: Characterization of the LRRK2-I1371V transfected U87 cell line; Figure S2: Immunocytochemistry (ICC) images of differentiated cells for mature neuronal marker MAP2, oligodendroglial marker O4 and microglial marker Cd11b; Figure S3: Whole blots for images used in the manuscript; Table S1: Research Resource Identifiers (RRID) of the antibodies used in the study; Table S2: Primers list.

## Abbreviations

PD—Parkinson’s disease; iPSC—induced pluripotent stem cells; HC—healthy control; LRRK2—leucine-rich repeat kinase 2; GFAP—glial fibrillary acidic protein; hPSCreg—Human Pluripotent Stem Cell Registry; FACS—fluorescence-activated cell sorting; ATP—adenosine triphosphate; SLC1A2—solute carrier family 1 member 2; SLC1A3—solute carrier family 1 member 3; ARM—armadillo; ANK—ankyrin; LRR—leucine-rich repeats; Roc—Ras of complex proteins; COR—C-terminal of Ros; GTP—guanosine triphosphate; DA—dopamine; Nrf2—nuclear factor erythroid-2-related factor 2; DNA—deoxyribonucleic acid; NF1A—nuclear factor 1A; AQP4—aquaporin 4; MAP2—microtubule-associated protein 2; MRP1—multidrug-resistance-associated protein 1; PCR—polymerase chain reaction; UV—ultraviolet; mRNA—messenger ribonucleic acid; SD—standard deviation; Oct4—octamer-binding transcription factor 4; SSEA4—stage-specific embryonic antigen-4; ; NPs—neural progenitors; EGF—epidermal growth factor; bFGF—basic fibroblast growth factor; GPCs—glial progenitor cells; CNTF—ciliary neurotrophic factor; BSA—bovine serum albumin; ICC—immunocytochemistry; GSH—reduced form of glutathione; GSSG—oxidized form of glutathione; GSS—glutathione synthetase; GR—glutathione reductase; GPx—glutathione peroxidase; GCL—glutamyl cysteine ligase; EV—cells transfected with empty vector plasmid; IV—cells transfected with LRRK2 I1371V mutation plasmid; cDNA—complementary DNA; TCA cycle—trichloro acetic acid cycle; GDH—glutamate dehydrogenase; NAD—nicotinamide adenine dinucleotide; SN1—system N1 Na^+^ and H^+^-coupled glutamine transporter; GS—glutamine synthase; ROS—reactive oxygen species; UPDRS—Unified Parkinson’s Disease Rating Scale; F-DOPA—fluoro-L-dopa; F-DOPA PET—fluoro-L-dopa positron emission tomography; NMDA receptor—N-methyl-D-aspartate receptor; w.r.t—with respect to.
