# Supplementary material for: Astrocytes Differentiated from LRRK2-I1371V Parkinson’s-Disease-Induced Pluripotent Stem Cells Exhibit Similar Yield but Cell-Intrinsic Dysfunction in Glutamate Uptake and Metabolism, ATP Generation, and Nrf2-Mediated Glutathione Machinery
Source: Cells. 2023 Jun 8;12(12):1592. doi: 10.3390/cells12121592 (PMC10297190; doi:10.3390/cells12121592)
Supplement: Supplementary file 1 [file cells-12-01592-s001.zip › cells-2345161-supplementary.pdf]

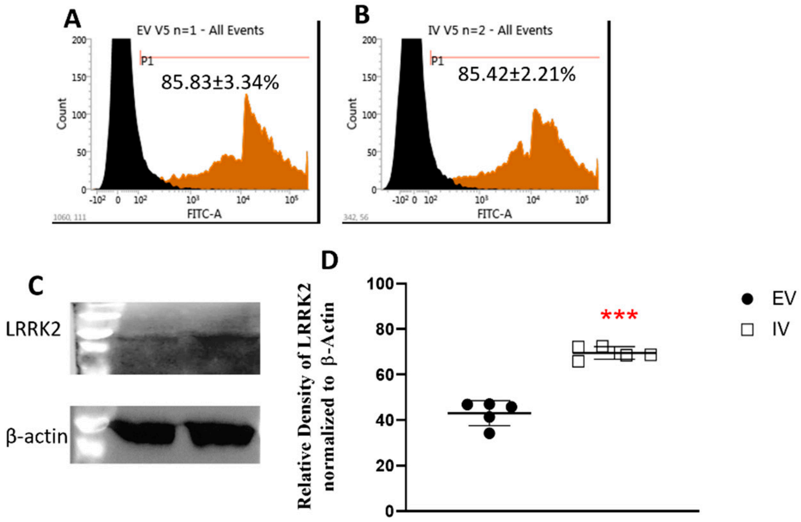

Supplementary Figure S1: (A,B) Representative FACS histogram of U87 cells transfected with empty vector (A) and U87 cells transfected with LRRK2 I1371V (B). Cells were identified by light scatter for 10,000 gated events. n=5. (C)Western blot bands obtained using antibodies against LRRK2 from 40µg of extracted cell lysate (EV: U87 cells transfected with EV, IV: U87 cells transfected with LRRK2 I1371V). (D) Densitometry plot of the bands obtained in western blot. Increased protein levels detected with IV cells [***$p < 0.001$], Student's t-Test result: $t = 5.6319$.

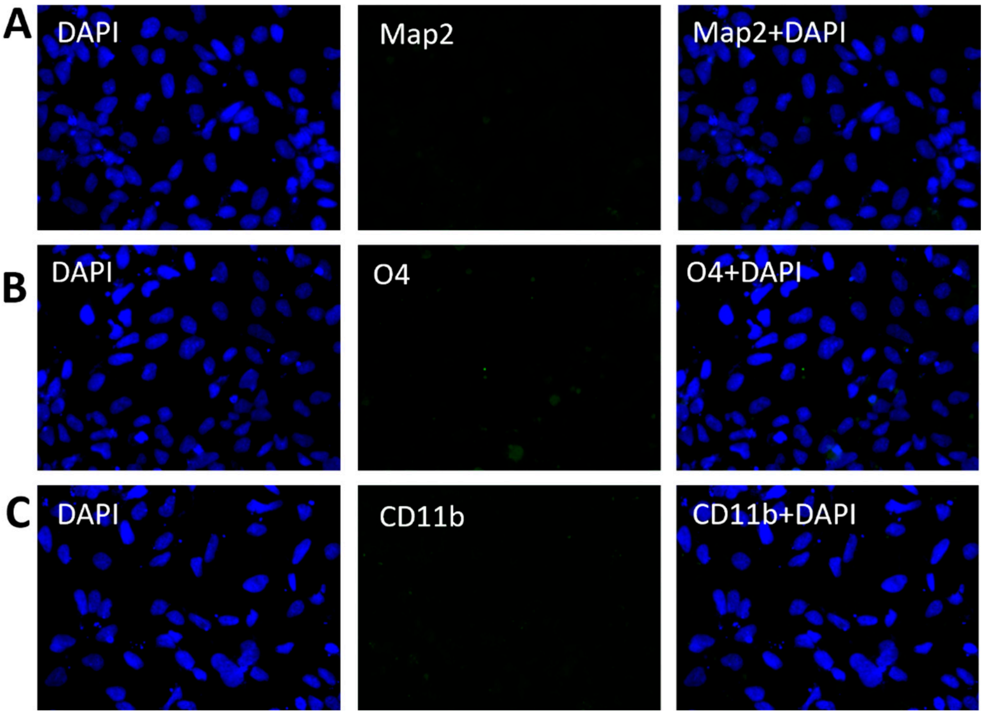

Supplementary Figure S2: Characterization of astrocytes for neuronal, oligodendrocyte and microglial markers: (A-C) Representative ICC images of astrocytes from HC and PD iPSC lines immunostained for neuronal marker MAP2 (A), oligodendrocyte marker O4 (B), microglial marker CD11b (C), expression.

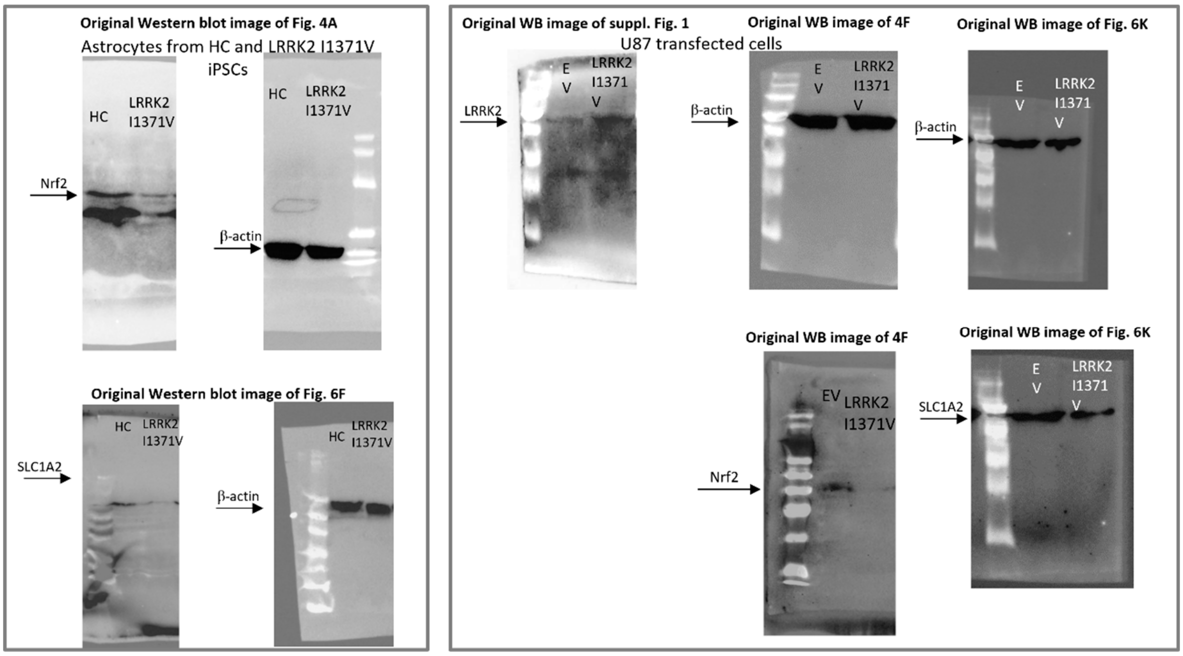

Supplementary Figure S3: Images of the whole western blots.

Supplementary Table S1: Research Resource Identifiers (RRID) and other details of the antibodies used in the study.

S. No.	Name (target)	RRID citation	Species raised in	Concentration used	Link to website
1	Anti-SSEA4 (SSEA4)	Thermo Fisher Scientific Cat# MA1-021, RRID:AB_2536687	Mouse	1:100 (Immunocyto-chemistry and Flow cytometry)	https://www.thermofisher.com/antibody/product/SSEA4-Antibody-clone-MC-813-70-Monoclonal/MA1-021

2	Anti-A2B5 (A2B5)	Innovative Research Cat# 433110, RRID:AB_1501794	Mouse	1:100 (Immunocyto-chemistry and Flow cytometry)	https://www.thermofisher.com/antibody/product/A2B5-Antibody-clone-105-Monoclonal/433110
3	Anti-NF1A (NF1A)	Thermo Fisher Scientific Cat# PA5-79735, RRID:AB_2746850	Rabbit	1:100 (Immunocyto-chemistry and Flow cytometry)	https://www.thermofisher.com/antibody/product/NFIA-Antibody-Polyclonal/PA5-79735
4	Anti-GFAP (GFAP)	Thermo Fisher Scientific Cat# PA5-16291, RRID:AB_10980769	Rabbit	1:100 (Immunocyto-chemistry and Flow cytometry)	https://www.thermofisher.com/antibody/product/GFAP-Antibody-Polyclonal/PA5-16291
5	Anti-SLC1A2 (SLC1A2)	Sigma-Aldrich Cat# WH0006506M10, RRID:AB_1841786	Mouse	1:100 (Immunocyto-chemistry and Flow cytometry)	https://www.sigmaaldrich.com/IN/en/product/sigma/wh0006506m10
6	Anti-Nanog (Nanog)	Abcam Cat# ab21624, RRID:AB_446437	Rabbit	1:100 (Immunocyto-chemistry and Flow cytometry)	https://www.abcam.com/nanog-antibody-ab21624.html
7	Recombinant Anti-Oct4 (Oct4)	Abcam Cat# ab21624, RRID:AB_446437	Rabbit	1:100 (Immunocyto-chemistry)	https://www.abcam.com/oct4-antibody-epr17929-chip-

					grade-ab181557.html
		Abcam Cat# ab27985, RRID:AB_776898	Goat	1:100 (Flow cytometry)	https://www.abcam.com/oct4-antibody-ab27985.html
8	Anti-TRA-1-60 (TRA-1-60)	Abcam Cat# ab16288, RRID:AB_778563	Mouse	1:100 (Immunocyto-chemistry and Flow cytometry)	https://www.abcam.com/tra-1-60-r-antibody-tra-1-60-ab16288.html
9	Recombinant Anti-Nestin (Nestin)	Abcam Cat# ab176571, RRID:AB_2895536	Rabbit	1:100 (Immunocyto-chemistry and Flow cytometry)	https://www.abcam.com/nestin-antibody-epr13012-ab176571.html
10	Recombinant Anti-Musashi1 (Musashi1 [Msi1])	Abcam Cat# ab52865, RRID:AB_881168	Rabbit	1:100 (Immunocyto-chemistry and Flow cytometry)	https://www.abcam.com/musashi-1--msi1-antibody-ep1302-ab52865.html
11	Anti-S100β (S100-A1)	Abcam Cat# ab868, RRID:AB_306716	Rabbit	1:100 (Immunocyto-chemistry and flow cytometry)	https://www.citeab.com/antibodies/760370-ab868-anti-s100-antibody-astrocyte-marker
12	Anti-Aquaporin (Aquaporin 4)	Abcam Cat# ab46182, RRID:AB_955676	Rabbit	1:100 (Immunocyto-chemistry and flow cytometry)	https://www.abcam.com/aquaporin-4-antibody-ab46182.html
13	Anti-MAP2	Thermo Fisher Scientific Cat# MA5-	Mouse	1:100 (Immunocyto-	https://www.thermofisher.com/antibody

		12823, RRID:AB_10982160		chemistry and flow cytometry)	/product/MAP2-Antibody-clone-AP20-Monoclonal/MA5-12823
14	Anti-CD11b	Millipore Cat# CBL1512, RRID:AB_93253)	Mouse	1:100 (Immunocyto-chemistry and flow cytometry)	https://www.merckmillipore.com/IN/en/product/Anti-Integrin-M-CD11b-Antibody-clone-OX-42,MM_NF-CBL1512
15	Anti-O4	(R and D Systems Cat# MAB1326, RRID:AB_357617)	Mouse	1:100 (Immunocyto-chemistry and flow cytometry)	https://www.rndsystems.com/products/human-mouse-rat-chicken-oligodendrocyte-marker-o4-antibody-o4_mab1326#product-datasheets
16	Anti-MRP1 (MRP1)	Abcam Cat# ab24102, RRID:AB_447868	Mouse	1:100 (flow cytometry)	https://www.abcam.com/mrp1-antibody-mrpm5-ab24102.html
17	Secondary antibody tagged with Alexa Fluor® 488 (IgG H&L)	Abcam Cat# ab150113, RRID:AB_2576208	Goat against mouse	1:200 (Immunocyto-chemistry and flow cytometry)	https://www.abcam.com/goat-mouse-igg-hl-alexa-fluor-488-ab150113.html<br><br>abcam.com/goat-rabbit-igg-hl-alexa-

		Abcam Cat# ab150077, RRID:AB_2630356	Goat against rabbit		fluor-488-ab150077.html
18	Secondary antibody tagged with Alexa Fluor® 647 (IgG H&L)	Abcam Cat# ab150115, RRID:AB_2687948<br><br>Abcam Cat# ab150075, RRID:AB_2752244	Goat against mouse<br><br>Donkey Goat against rabbit	1:200 (Immunocyto-chemistry and flow cytometry)	https://www.abcam.com/goat-mouse-igg-hl-alexa-fluor-647-ab150115.html<br><br>https://www.abcam.com/donkey-rabbit-igg-hl-alexa-fluor-647-ab150075.html
19	Anti-β-actin (β-actin)	(Abcam Cat# ab8227, RRID:AB_2305186)	Mouse	1:1000 (Western blot)	https://www.abcam.com/beta-actin-antibody-ab8227.html
20	HRP conjugated secondary antibody (IgG H&L)	Abcam Cat# ab97051, RRID:AB_10679369<br><br>Abcam Cat# ab97023, RRID:AB_10679675	Goat against rabbit<br><br>Goat against mouse	1:2000 (Western blot)	https://www.abcam.com/goat-rabbit-igg-hl-hrp-ab97051.html<br><br>https://www.abcam.com/goat-mouse-igg-hl-hrp-ab97023.html
21	Anti-Vimentin antibody (Vimentin)	BD Biosciences Cat# 550513, RRID:AB_393716	Mouse	1:100 (Immunocyto-chemistry and flow cytometry)	https://www.bdbiosciences.com/ko-kr/products/reagents/microscopy-imaging-reagents/purified-mouse-anti-vimentin.550513

22	Anti-Nrf2 antibody (Nrf2)	Santa Cruz Biotechnology Cat# sc-365949, RRID:AB_10917561	Rabbit	1:1000 (Western blot) 1:100 (Immunocyto-chemistry)	https://www.scbt.com/p/nrf2-antibody-a-10
23	Anti-V5 tag antibody	Thermo Fisher Scientific Cat# MA1-34099, RRID:AB_1959285	Mouse	1:100 (Immunocyto-chemistry)	https://www.thermofisher.com/antibody/product/V5-Tag-Antibody-clone-SV5-Pk1-Monoclonal/MA1-34099
24	Anti-LRRK2 antibody	Thermo Fisher Scientific Cat# PA5-18319, RRID:AB_10988665	Goat	1:1000 (Western blot)	https://www.thermofisher.com/antibody/product/LRRK2-Antibody-Polyclonal/PA5-18319

Supplementary Table S2: Primer list

Gene name	Forward/ Reverse	Sequence (5' – 3')
18S	Forward	CGGCTACCACATCCAAGGAA
	Reverse	GCTGGAATTACCGCGGCT
CD44	Forward	TTACACCTTTTCTACTGTACACCCC
	Reverse	TCAGATCCATGAGTGGTATGGGAC
Nuclear factor 1 A-type (NF1A)	Forward	GACTTCTGGCAAAGTTGCGG
	Reverse	TCGCATCTTGCCTTTCTGGT
Glial fibrillary acidic protein (GFAP)	Forward	ATCGAGAAGGTTCGCTTCCTG
	Reverse	TGTTGGCGGTGAGTTGATCG
S100β	Forward	GGAGACAAGCACAAGCTGAAG
	Reverse	AGCTACAACACGGCTGGAAAG
Aquaporin 4 (AQP4)	Forward	GGAATCCTCTATCTGGTCACA
	Reverse	TGTTTGCTGGGCAGCTTTGCT
Glutamine synthase (GS)	Forward	CTGCCATACCAACTTCAGCACC
	Reverse	ATAGGCACGGATGTGGTACTGG

System N amino acid transporter-1 (SN1)	Forward	GCCACTTGTCATACAGACCTTCC
	Reverse	GGCAGAATGATGGTGACAGAGAC
Solute carrier family 1, member 2 (SLC1A2)	Forward	TGCCAACAGAGGACATCAGCCT
	Reverse	CAGCTCAGACTTGGAGAGGTGA
Solute carrier family 1, member 3 (SLC1A3)	Forward	GGTTGCTGCAAGCACTCATCAC
	Reverse	CACGCCATTGTTCTCTTCCAGG
Glutamate dehydrogenase (GDH)	Forward	CTCCAGACATGAGCACAGGTGA
	Reverse	CCAGTAGCAGAGATGCGTCCAT
Glutathione synthetase (GSS)	Forward	CCAAGACCGAAGGCTGTTTGTG
	Reverse	TGTGACCTCTCCAGCAGTAGAC
Glutathione peroxidase (GPx)	Forward	TTCGAGCCCAACTTCATGCT
	Reverse	CGATGTCAGGCTCGATGTCA
Glutathione reductase (GR)	Forward	TATGTGAGCCGCCTGAATGCCA
	Reverse	CACTGACCTCTATTGTGGGCTTG
Glutamyl cysteine ligase C (GCLC)	Forward	GGAAGTGGATGTGGACACCAGA
	Reverse	GCTTGTAGTCAGGATGGTTTGCG
